# A Case Highlighting the Importance of Timely Intervention for a Male With AZFb + c Microdeletion

**DOI:** 10.1155/crig/2832648

**Published:** 2026-05-21

**Authors:** Rahul Atodaria, Randa Abdelmasih

**Affiliations:** ^1^ Department of Internal Medicine, University of Texas Medical Branch, Galveston, Texas, USA, utmb.edu; ^2^ Department of Internal Medicine, Division of Endocrinology, University of Texas Medical Branch, Galveston, Texas, USA, utmb.edu

**Keywords:** AZF, azoospermia, infertility, microdeletion

## Abstract

Male infertility can be caused by various microdeletions of the azoospermia factor (AZF) gene, some of which require sperm retrieval techniques for treatment. Yet, the likelihood of success with such techniques depends partly on the affected AZF gene segment. For patients with select AZF microdeletions, other options such as in vitro fertilization, sperm donation, and adoption may be more appropriate. We present a case about a 32‐year‐old male diagnosed with infertility due to azoospermia from a complete AZFb + c deletion, which is unlikely to be successfully treated with sperm retrieval techniques. The goal of this case report is to highlight the significance of earlier diagnosis of AZF microdeletions in infertile males to facilitate earlier genetic counseling and pursuit of the most appropriate intervention.

## 1. Introduction

Fifty percent of all infertility cases are due to male infertility [1–6]. Among the various etiologies of infertility that can affect males—such as medications, congenital conditions, and obstructive pathology—acquired or genetic testicular failure is the most common cause. Microdeletions of the Y chromosome’s azoospermia factor (AZF) gene are one of the major genetic causes of testicular failure. These microdeletions are the second most common genetic cause of male infertility (behind Klinefelter syndrome) and are seen in at least 1 out of every 4000 males in the general population [6–9]. Among males with azoospermia or severe oligozoospermia, the frequency of AZF microdeletions ranges at least between 5%–12% and 2%–7%, respectively [2, 9–11]. Furthermore, the global prevalence of AZF microdeletions among infertile males is 7% [1, 12, 13]. Treatment of azoospermia in patients with AZF gene microdeletions requires microdissection testicular sperm extraction (mTESE) and intracytoplasmic sperm injection (ICSI), which can have varying success rates depending on which segment of the AZF gene is deleted [2, 3, 7].

## 2. Case Presentation

### 2.1. History

A 32‐year‐old male with no chronic medical conditions, including diabetes mellitus, liver disease, and obesity, or relevant family history presented for evaluation of an inability to have biological children despite unprotected intercourse for three years with his healthy 29‐year‐old female partner. He denied impairment of energy levels, libido, physical performance, sleep habits, mood, and abnormalities in his sense of smell. As a teenager, he had experienced trauma to his right testicle from a baseball. Otherwise, he did not have major lifetime illnesses, including mumps. He had also never undergone abdominal or genitourinary surgeries. He denied any known exposure to radiation, lead, or hormonal therapy. He had met appropriate puberty‐related milestones. He smokes one‐half packs of cigarettes per week and drinks between five to ten alcoholic drinks per week. He previously worked as a merchant marine. He does not take any medications. The patient had never fathered any biological children. His partner had been diagnosed with ovarian cysts and had previously been pregnant from another partner around age 18, which ended in spontaneous abortion.

### 2.2. Diagnostic Assessment

His physical examination, including inspection of his genitalia, was notable for right testicular atrophy. There were no signs of gynecomastia. Vital signs were normal. Prior to presentation, he had undergone semen analysis and scrotal ultrasound by Urology. Semen analysis revealed azoospermia. Scrotal ultrasound showed small bilateral hydroceles and a left varicocele. Urology discussed that varicoceles in general can be associated with infertility but were unlikely to be the sole cause for this patient given his azoospermia.

Based on this information, we obtained a comprehensive hormone analysis to investigate for primary and secondary hypogonadism as the etiology of azoospermia. The findings are summarized in Tables 1 and 2. The low total testosterone level was at least partly due to the low sex hormone–binding globulin level, especially when considering he did not exhibit clear symptoms of hypogonadism. Because of the elevated prolactin level, a brain MRI was obtained to investigate for a possible hormone‐secreting tumor or other structural abnormalities of the hypothalamus and pituitary gland. However, the brain MRI did not reveal any acute or chronic abnormalities that correlated with the laboratory results. Then, because of the elevated FSH and LH levels and possible low total testosterone levels, we strongly suspected primary testicular failure as the cause of infertility.

**TABLE 1:** Summary of laboratory data.

Hormone	Measured value	Reference range
Total testosterone	263 ng/dL (9.13 nmol/L)	300–1080 ng/dL (10.4–37.5 nmol/L)
	Repeat 204 ng/dL (7.08 nmol/L)	300–1080 ng/dL (10.4–37.5 nmol/L)

Free testosterone	68 pg/mL	47–244 pg/mL

Sex‐hormone‐binding globulin	13 nmol/L	17–56 nmol/L

Estradiol	23 pg/mL	20–50 pg/mL

Follicle‐stimulating hormone	26.4 mIU/mL	1.50–12.4 mIU/mL

Luteinizing hormone	14.3 mIU/mL	1.20–7.80 mIU/mL

Prolactin	25.3 ng/mL	2.60–13.1 ng/mL

**TABLE 2:** Summary of laboratory data, continued.

Item	Measured value	Reference range
Hemoglobin A1C	5.4%	< 5.7%
Total cholesterol	278 mg/dL	120–200 mg/dL
Triglycerides	312 mg/dL	30–170 mg/dL
High‐density lipoprotein (HDL)	66 mg/dL	> 40 mg/dL
Low‐density lipoprotein (LDL)	150 mg/dL	< 160 mg/dL
Very‐low‐density lipoprotein (VLDL)	62 mg/dL	5–60 mg/dL
Alanine aminotransferase (ALT)	85 U/L	5–50 U/L
Aspartate aminotransferase (AST)	54 U/L	13–40 U/L
Alkaline phosphatase (ALP)	51 U/L	34–122 U/L

**FIGURE 1:**
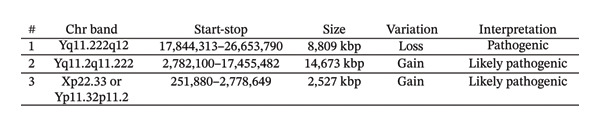
Pathogenic and likely pathogenic mutations identified on chromosome microarray.

To evaluate for genetic etiologies of primary testicular failure, such as Klinefelter syndrome, chromosome microarray testing was performed using the Affymetrix CytoScan HD microarray. There were three mutations that were identified (Figure 1). First, an 8.8 Mb hemizygous loss was found at Yq11.222q12, which included the complete AZFb + c segment. Next, there were two gain mutations on the Y chromosome. Of the two, the 14.7 Mb gain at Yp11.2q11.222 represented a duplication of the AZFa segment which can be observed in people with developmental delays (not present in our patient). Collectively, these mutations indicate that a pseudo dicentric Y chromosome was present, created from at least a partial deletion of the Y chromosome’s long arm and duplication of the short arm.

### 2.3. Treatment

Based on the chromosomal analysis results, we discussed with the patient that the likelihood of having a child naturally would be very low and that fertility treatments would most likely be needed. For his hypogonadism, we did not recommend testosterone therapy at that time due to lack of symptoms of testosterone deficiency. Prior to the patient’s presentation to our Endocrinology clinic, Urology recommended surgical repair of his varicocele as a potential management step for his infertility. Ultimately, the patient elected to revisit the option of surgical repair of the varicocele with Urology considering these genetic findings before deciding whether he wanted to pursue fertility treatments. A referral to Medical Genetics was provided for more detailed counseling regarding the chromosomal microarray results. He was counseled that the likelihood of having biological children would be low and that any of his sons would likely inherit the same microdeletion. The possibility of genetic screening for aneuploidy and sex selection prior to implantation was discussed. He and his wife elected to discuss reproductive options, including in vitro fertilization, sperm donation, and adoption with the Reproductive Endocrinology clinic.

### 2.4. Outcome and Follow‐Up

To date, the patient had not yet followed up with Urology or with our Endocrinology clinic. He had also not yet established care with the Reproductive Endocrinology clinic. Unfortunately, this had prevented discussion regarding the need for further karyotype and fluorescence in situ hybridization (FISH) to identify the evidence of mosaicism.

## 3. Discussion

We present a case of male infertility due to azoospermia from a complete AZFb + c microdeletion of the Y chromosome. In general, AZF microdeletions are rare in the general population yet convey a significant emotional and psychological burden onto affected patients. Furthermore, the most appropriate treatment approach for this type of infertility depends on the specific AZF microdeletion as each carries a different prognosis. Therefore, the goal of this case report is to emphasize the significance of earlier diagnosis of AZF microdeletions as to facilitate earlier genetic counseling as well as the identification and pursuit of the most appropriate intervention.

For instance, AZFc deletions are the most common subtype and manifest as the least severe phenotype. With experienced surgeons and embryologists, mTESE can successfully obtain testicular sperm in as many as 50% of the patients with AZFc deletions [2, 6]. A study by Huang even showed a mTESE success rate of 74.5% based on 47 males with azoospermia from AZFc microdeletions [7].

On the other hand, AZFa and AZFb deletions are associated with poor likelihood (near 0%) of successful sperm retrieval due to a complete lack of germ cells or maturation arrest of germ cells, respectively. As such, sperm injection and sperm extraction techniques are typically contraindicated in patients with AZFa or AZFb microdeletions due to very high failure rates [2–4, 6, 13, 14].

With less severe microdeletions, such as the partial AZFb + c deletion, affected patients can exhibit hypospermatogenesis, which may leave a reasonable chance of successful sperm retrieval. A study by Kleiman et al. found that the chance of successful sperm retrieval was higher in males with partial b + c deletions compared with males with complete b + c deletions (43% vs. 3%, *p* < 0.0001) [15]. Krausz et al. discussed the importance of performing deletion extension analysis in such patients to determine if there is any residual spermatogenesis and if mTESE should be attempted [6]. Specifically, deletion extension analysis is a method of more precisely defining the extent of a DNA deletion by utilizing a larger number of genetic markers.

Unfortunately, even in the minority of cases involving successful sperm retrieval from males with complete b + c deletions, there have not been any successful pregnancies after ICSI [15]. This means that sperm retrieval is not a viable option for these patients. Focus must shift toward in vitro fertilization, sperm donation, or adoption. Consequently, for couples consisting of an affected male and his female partner, maternal age becomes the primary determining factor regarding the likelihood of success with assisted reproductive therapies. In fact, the probability of a live birth from a single in vitro fertilization attempt was shown to be 43.1% for women younger than 35 years of age, which then continued to decrease to 3.2% for women older than 42 years of age [16]. This reiterates the importance of timeliness in diagnosis.

In conclusion, we recommend endocrinologists maintain a low threshold for initiating genetic analysis in infertile males as earlier diagnosis would lead to earlier genetic counseling as well as earlier determination and pursuit of the most appropriate therapeutic intervention. For instance, earlier diagnosis of AZFb deletions can facilitate more timely discussion about genetic counseling and alternative routes to having children, considering that any male offspring of males with AZF microdeletions would inherit the AZF deletion [9]. Couples may even need to consider preimplantation genetic testing to select for female embryos or use donor sperm for conception. For males with AZFc deletions, earlier diagnosis would counter age‐related decline in the quality and quantity of sperm and possibly optimize mTESE/ICSI outcomes.

## Funding

No public or commercial funding was involved in this project.

## Disclosure

This abstract was submitted in partially completed state and accepted for poster presentation at ENDO Society meeting on July 12, 2025, in San Francisco, California.

## Consent

Signed informed consent was obtained directly from the patient.

## Conflicts of Interest

The authors declare no conflicts of interest.

## Data Availability

Data sharing is not applicable to this article as no datasets were generated or analyzed during the current study.
